# Targeted Atherosclerosis Treatment Using Vascular Cell Adhesion Molecule-1 Targeting Peptide-Engineered Plant-Derived Extracellular Vesicles

**DOI:** 10.3390/ijms26188884

**Published:** 2025-09-12

**Authors:** Chanwoo Choi, Won Jong Rhee

**Affiliations:** 1 Department of Bioengineering and Nano-Bioengineering, Incheon National University, Incheon 22012, Republic of Korea; woochoi9797@gmail.com; 2 Division of Bioengineering, Incheon National University, Incheon 22012, Republic of Korea; 3 Research Center for Bio Materials & Process Development, Incheon National University, Incheon 22012, Republic of Korea

**Keywords:** atherosclerosis, extracellular vesicle, onion, VHPK peptide, VCAM-1

## Abstract

Atherosclerosis is a chronic vascular disease characterized by lipid accumulation, endothelial dysfunction, and persistent inflammation, which can ultimately lead to life-threatening complications, such as myocardial infarction and stroke. Current therapies primarily focus on lowering cholesterol levels or preventing blood clot formation. However, the multifactorial and dynamic nature of atherosclerotic progression is not addressed. We designed a therapeutic platform based on onion-derived extracellular vesicles (Onex), nanovesicles originating from onions with excellent biocompatibility and strong anti-inflammatory effects. Onex was engineered with the VHPK peptide, to construct V-Onex, specifically targeting vascular cell adhesion molecule-1 (VCAM-1), which is strongly upregulated in inflamed endothelial cells during atherosclerosis. Engineered V-Onex exhibited excellent biocompatibility and stability without inducing cytotoxicity in human umbilical vein endothelial cells (HUVECs) and THP-1 cells. V-Onex selectively accumulated in inflamed endothelial cells and significantly reduced the expression of inflammatory markers in HUVECs and THP-1 cells. It also suppresses the migration of endothelial cells and reduces their interaction with monocytes, both of which contribute to plaque formation. In THP-1 cells, V-Onex inhibited the uptake of oxidized low-density lipoprotein and reduced foam cell formation. Collectively, V-Onex is a promising modular targeted nanovesicle platform capable of modulating multiple pathological processes associated with atherosclerosis.

## 1. Introduction

Cardiovascular disease (CVD) remains the leading cause of death worldwide, accounting for approximately one-third of all deaths. As the population ages, the burden of CVD is expected to increase, because age-related changes, such as vascular inflammation and lipid imbalance, contribute to atherosclerosis [1,2]. Atherosclerosis, a chronic inflammatory condition of the arterial wall, is widely recognized as the core mechanism of CVD and leads to serious events, such as myocardial infarction and stroke [3,4]. Inflammatory stimuli trigger endothelial dysfunction, which induces the expression of adhesion molecules and chemokines that recruit circulating monocytes to the vascular intima. These monocytes differentiate into macrophages and internalize oxidized low-density lipoprotein (oxLDL), resulting in foam cell formation [5,6,7]. Progressive foam cell accumulation leads to plaque growth and instability, which disrupts vascular integrity and promotes thrombotic complications [8,9,10]. Among the adhesion molecules, vascular cell adhesion molecule-1 (VCAM-1) plays a critical role in monocyte recruitment. It is highly expressed at lesion sites and facilitates the binding between endothelial cells and monocytes. This interaction not only enhances immune cell infiltration, but also amplifies local inflammation and accelerates plaque development [11,12,13,14,15]. Owing to its localized and pathological relevance, VCAM-1 is considered a promising target for the delivery of anti-atherosclerotic therapeutics [16,17,18,19,20]. Current therapies for atherosclerosis largely focus on lowering cholesterol using statins, which reduce cardiovascular risk by lowering LDL levels [21,22,23,24,25]. However, these studies did not effectively address the initiating mechanisms of atherosclerosis, such as chronic vascular inflammation, endothelial activation, and immune cell recruitment. Moreover, administered drugs are distributed systemically, reaching non-diseased tissues and potentially causing adverse effects, such as myalgia and hepatotoxicity [26,27,28]. Such off-target distributions, along with their limited efficacy, underscore the need for lesion-targeted therapeutics capable of selectively acting on pathological sites while simultaneously modulating multiple disease mechanisms [29,30].

Extracellular vesicles (EVs) are nanosized carriers secreted by cells that transport bioactive molecules and modulate cellular responses [31,32]. Plant-derived EVs have recently emerged as attractive alternatives to mammalian EVs because of their low immunogenicity, cost-effectiveness, and ease of production [33,34,35,36]. They can be isolated from edible tissues without the need for bioreactors, allowing for scalable and safe manufacturing. Moreover, some plant EVs display natural tissue-targeting abilities and efficient uptake. These favorable features contrast with synthetic nanocarriers such as liposomes and polymeric nanoparticles, which often suffer from immunogenicity, rapid clearance, and manufacturing complexity [37]. However, their therapeutic applications remain underexplored and the lack of standardization has slowed their clinical translation [38,39,40,41,42,43].

**Scheme 1:**
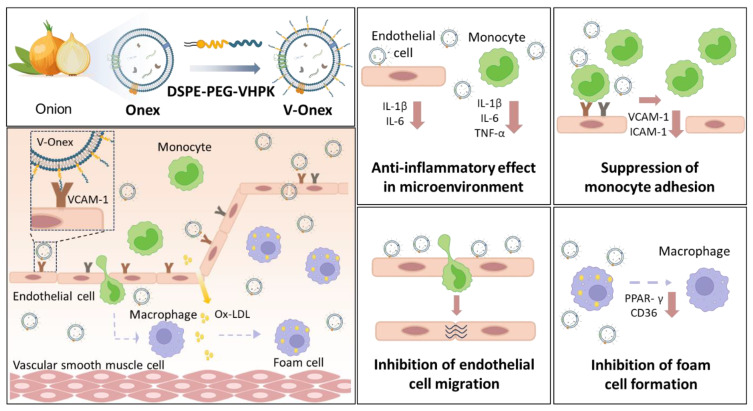
Construction of engineered onion-derived extracellular vesicles (V-Onex) for VCAM-1-targeted atherosclerosis therapy. Ox-LDL, oxidized low-density lipoprotein.

Among plant-derived EVs, onion-derived EVs (Onex) provide an additional advantage, as our previous study demonstrated that Onex suppressed inflammation in LPS-stimulated macrophages by downregulating pro-inflammatory mediators (IL-6, IL-1β, COX-2, iNOS) via NF-κB modulation, without cytotoxicity even at high concentrations. Moreover, Onex can be obtained with high yield and low cost using SEC combined with ultrafiltration, demonstrating its practicality for reproducible and scalable production [44]. This study aimed to evaluate the anti-atherosclerotic potential of Onex. VCAM-1 plays a pivotal role in the early stages of lesion formation and is markedly overexpressed at atherosclerotic sites; therefore, a VCAM-1-binding peptide was conjugated to Onex using DSPE-PEG to generate V-Onex to improve lesion targeting. V-Onex was investigated in human umbilical vein endothelial cells (HUVECs) and THP-1 monocytes, and showed enhanced suppression of inflammation, migration, and monocyte adhesion, particularly in inflamed endothelial cells (Scheme 1). Therefore, this study was designed to investigate the therapeutic potential of V-Onex by evaluating its effects on vascular inflammation and key processes underlying atherosclerosis.

## 2. Results and Discussion

### 2.1. Characterization and Cytotoxicity Assessment of Onex

**Figure 1:**
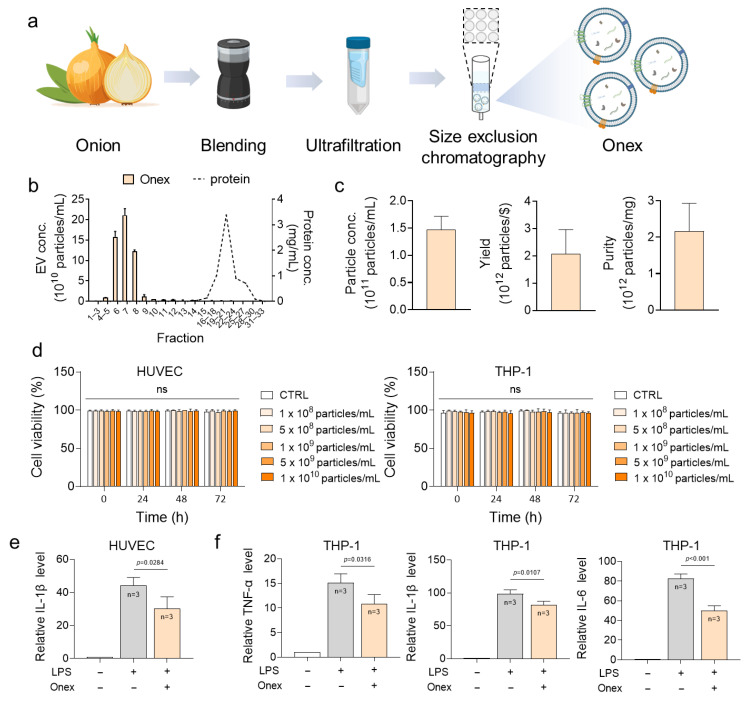
Isolation and characterization of onion-derived extracellular vesicles (Onex). (**a**) Schematic illustration of the Onex isolation process involving blending, ultrafiltration, and size-exclusion chromatography. (**b**) Distribution profiles of EV and protein concentrations in SEC fractions of onion extract. (**c**) Particle concentration was determined using nanoparticle tracking analysis (NTA), while yield was expressed as particles per US dollar of production cost, and purity was calculated based on particle count and protein content. (**d**) Cell viability of HUVECs and THP-1 cells after 24, 48, and 72 h of Onex treatment at various concentrations (1 × 10^8^ to 1 × 10^10^ particles/mL). (**e**) Expression levels of IL-1β in HUVECs analyzed to evaluate the anti-inflammatory effect of Onex. (**f**) Expression levels of IL-1β, IL-6, and TNF-α in THP-1 cells analyzed to evaluate the anti-inflammatory effect of Onex. Data are presented as mean ± SEM. Statistical significance was determined using one-way ANOVA. EV, extracellular vesicle; HUVEC, human umbilical vein endothelial cell.

Onex was isolated from *Allium cepa* through a sequential purification process involving ultrafiltration and size-exclusion chromatography. Fresh onions were thoroughly washed, diced, and the juice was extracted using a mechanical juicer. The crude extract was subjected to sequential centrifugation at 8000× *g* and 20,000× *g* for 1 h each to eliminate debris and large particles. The clarified supernatant was concentrated to 500 μL using centrifugal filter units (Amicon Ultra-15, Merck Millipore, Darmstadt, Germany) at 5000× *g*, and subsequently purified through SEC (Izon Science, Christchurch, New Zealand), yielding 33 fractions (Figure 1a). According to NTA profiles and protein measurements, vesicles were highly enriched in fractions 6–8, while most free proteins eluted in later fractions. These vesicle-rich fractions were pooled and designated as Onex, which was stored at −80 °C until further use (Figure 1b). This sequential purification process effectively minimized protein contamination and maintained vesicle integrity, thereby establishing a reliable isolation protocol.

To characterize the physicochemical properties of Onex, NTA was conducted. Onex exhibited a consistently high concentration, with an average particle concentration of 1.45 × 10^11^ particles/mL. The yield was 2.0 × 10^12^ particles per US dollar of production cost, reflecting the cost-efficiency of the isolation process. The purity reached 2.1 × 10^12^ particles per mg protein, indicating that the vesicle population was largely free from protein contamination. These results demonstrate the reproducibility and scalability of the isolation protocol (Figure 1c) and suggest that the isolated vesicles have a size distribution and purity that meet the criteria for potential nanotherapeutic applications.

To evaluate the cytotoxicity of Onex, HUVECs and THP-1 cells were treated with various concentrations of Onex (1 × 10^8^–1 × 10^10^ particles/mL) for 72 h. Cell viability was measured using the trypan blue assay. The results showed no cytotoxic effects on either cell type, even at the highest concentration, confirming the high biocompatibility of Onex (Figure 1d). The absence of cytotoxicity across a wide dose range supports the feasibility of using Onex in future applications.

### 2.2. Evaluation of the Anti-Inflammatory Activity of Onex in HUVECs and THP-1 Cells

To assess the anti-inflammatory effects of Onex, LPS-induced HUVECs were treated with Onex. HUVECs were stimulated with LPS to induce inflammation, followed by treatment with Onex (1 × 10^10^ particles/mL). qRT-PCR analysis showed that IL-1β expression, which was highly induced by LPS in the absence of Onex, was significantly reduced in the presence of Onex (Figure 1e). Specifically, IL-1β expression levels were reduced by 31% by Onex treatment. In addition to endothelial cells, anti-inflammatory effects of Onex have also been observed in macrophages. Similarly, THP-1 cells that differentiated into M0 macrophages using PMA (Phorbol 12-myristate 13-acetate) were stimulated with LPS. Onex attenuated the LPS-induced expression of pro-inflammatory cytokines in these macrophages, reducing TNF-α, IL-1β, and IL-6 by 17, 17, and 39%, respectively (Figure 1f). The consistent downregulation of inflammatory markers in both endothelial cells and macrophages highlighted the broad anti-inflammatory effects of Onex. It is particularly encouraging that the anti-inflammatory function of Onex is not confined solely to endothelial cells, but also extends to macrophages, which are key players in the progression of atherosclerosis at sites of inflammation. These characteristics collectively highlight the potential of Onex as a plant-derived EV formulation capable of modulating inflammatory pathways associated with atherosclerosis, thereby supporting its applicability as a foundational nanovesicle platform for targeted therapeutic interventions in vascular inflammation.

### 2.3. V-Onex Construction by Surface Engineering of Onex and Their Characterization

**Figure 2:**
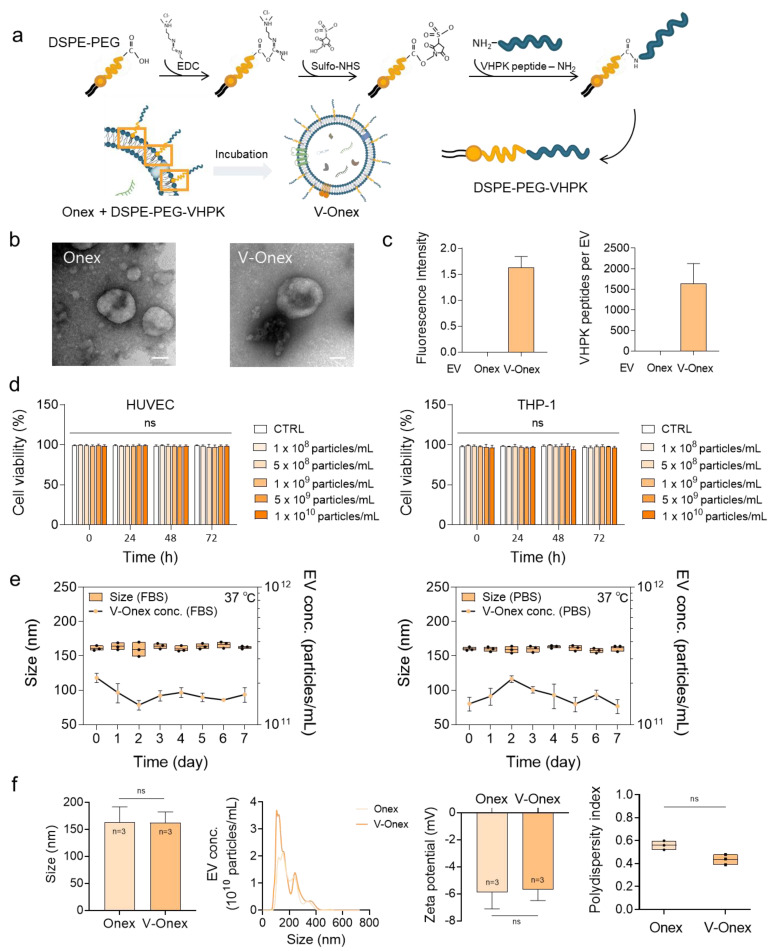
Construction and characterization of VHPK-engineered vesicles (V-Onex). (**a**) Schematic illustration of the synthesis of DSPE-PEG-VHPK conjugates via EDC/NHS chemistry. (**b**) Representative TEM images of Onex and V-Onex showing preserved vesicle morphology. Scale bar = 50 nm. (**c**) Quantification of surface-conjugated VHPK peptides on V-Onex based on FITC fluorescence intensity and NTA particle count. (**d**) Cell viability of HUVECs and THP-1 cells after treatment with V-Onex at concentrations ranging from 1 × 10^8^ to 1 × 10^10^ particles/mL for 24, 48, and 72 h, measured using the trypan blue assay. (**e**) Size and particle concentration of V-Onex incubated in PBS or PBS containing 10% FBS at 37 °C over 7 days. (**f**) Zeta potential, size, particle concentration, and PDI of Onex and V-Onex. Data are presented as mean ± SEM. Black dots represent individual experimental data points. Statistical significance was determined using one-way ANOVA: ns: not significant. EV, extracellular vesicle; FBS, fetal bovine serum; HUVEC, human umbilical vein endothelial cell; NTA, nanoparticle tracking analysis.

Targeting inflammatory endothelial cells is necessary to efficiently control inflammation within an in vivo vascular environment. To enable the targeted therapeutic delivery of Onex to the inflamed endothelium, the VHPK peptide was incorporated onto the surface of Onex using a DSPE-PEG-based insertion strategy to construct V-Onex. The VHPK peptide, which specifically binds to VCAM-1, was first conjugated to DSPE-PEG-COOH via (1-(3-dimethylaminopropyl)-3-ethylcarbodiimide) (EDC)/N-hydroxysulfosuccinimide sodium salt (NHS) coupling. The resulting DSPE-PEG-VHPK was then spontaneously inserted into the lipid bilayer of Onex via hydrophobic interactions between the DSPE lipid tail and EV membrane (Figure 2a). This membrane-anchoring approach provides a simple and non-disruptive method for engineering surface ligands onto EVs without altering their core structure.

The structural morphology of the engineered V-Onex was evaluated using TEM (Transmission Electron Microscopy, JEM-1010, JEOL Ltd., Tokyo, Japan). The images revealed that V-Onex retained a characteristic cup-shaped morphology, which is consistent with the typical structure of EVs (Figure 2b). The preservation of the structural features suggests that the surface engineering procedure does not compromise the vesicle architecture. To confirm the successful incorporation of VHPK onto the vesicle surface, FITC-labeled VHPK was used and the fluorescence intensity was quantified after removing free VHPK. V-Onex prepared using FITC-VHPK exhibited strong fluorescence, whereas native Onex showed no detectable signal. A standard calibration curve generated using free FITC-VHPK enabled estimation of the number of peptides per vesicle. The results indicated that each V-Onex particle contained approximately 1500 peptides (Figure 2c). The formulation was optimized to avoid micelle formation and ensure uniform peptide incorporation (Figure S1).

To evaluate the cytotoxic potential of V-Onex, HUVEC and THP-1 cells were treated with increasing concentrations of V-Onex ranging from 1 × 10^8^ to 1 × 10^10^ particles/mL for 72 h. No significant reduction in viable cells was observed under any of the tested conditions, indicating that engineered V-Onex did not induce cytotoxicity in either cell line (Figure 2d). This finding is particularly important for therapeutic applications, as it confirms that peptide modification does not induce acute cellular toxicity even at high particle concentrations.

Storage and serum stability of V-Onex were assessed by incubating vesicles in PBS and PBS supplemented with 10% fetal bovine serum (FBS) at 37 °C for seven days. In PBS, the particle size remained stable at approximately 160 nm and the particle concentration showed no significant decline. Similarly, in PBS containing 10% serum, both the size and concentration were well preserved, confirming the stability of V-Onex under physiological conditions (Figure 2e). This result indicates that V-Onex can retain its physicochemical integrity even in protein-rich biological fluids, which is critical for its systemic administration. Further characterization using NTA confirmed that the average particle size of V-Onex was approximately 160 nm, similar to that of native Onex. The zeta potential and polydispersity index (PDI) values were also comparable between V-Onex and unmodified Onex, indicating that surface modification did not alter the vesicle dispersity or surface charge properties (Figure 2f). The ability to maintain colloidal stability following functionalization underscores the robustness of this engineering approach. These results demonstrated that VHPK was effectively introduced onto the surface of Onex using a simple membrane insertion method. This non-invasive technique eliminates the need for harsh solvents or mechanical extrusion, thereby preserving the structure and function of EVs. Moreover, the fact that the engineered vesicles retained their physicochemical properties, including size distribution, colloidal stability, and surface charge, highlighted the robustness of this surface functionalization platform.

### 2.4. Targeting Capability of V-Onex in Inflamed Endothelial Cells

**Figure 3:**
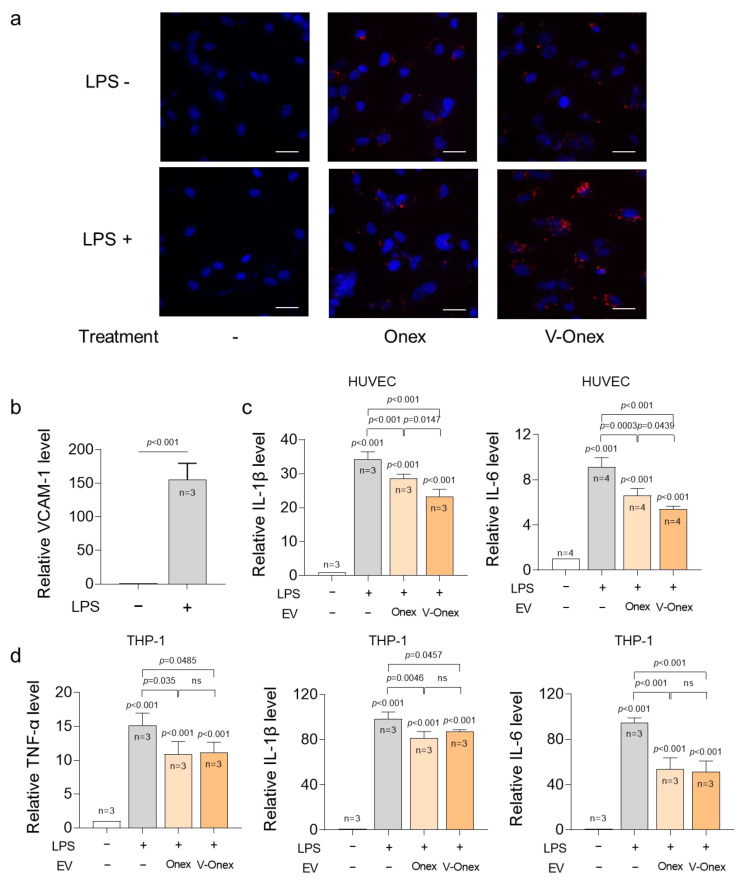
Targeting capability of V-Onex in endothelial cells and anti-inflammatory effect in HUVECs and THP-1 cells. (**a**) Fluorescence microscopy images showing cellular uptake of DiI-labeled Onex and V-Onex in HUVECs with or without LPS stimulation with size bars indicating 50 μm. (**b**) Relative VCAM-1 mRNA expression in HUVECs showing significant upregulation upon LPS stimulation, assessed using qRT-PCR. (**c**) Relative mRNA expression levels of IL-1β and IL-6 in LPS-stimulated HUVECs treated with Onex or V-Onex, analyzed using qRT-PCR. (**d**) Relative mRNA expression levels of TNF-α, IL-1β and IL-6 in LPS-stimulated THP-1 cells treated with Onex or V-Onex, analyzed using qRT-PCR. Data are presented as mean ± SEM. Statistical significance was determined using one-way ANOVA: ns: not significant. EV, extracellular vesicle; HUVEC, human umbilical vein endothelial cell; LPS, lipopolysaccharide.

To validate the targeting capability of V-Onex under inflammatory conditions, its interaction with activated endothelial cells was evaluated using fluorescence microscopy. Onex and V-Onex were labeled with the lipophilic dye DiI. All the samples were applied at a concentration of 1 × 10^10^ particles/mL because there was no toxicity observed with this concentration (Figure 2d). HUVEC nuclei were stained with DAPI for visualization (Figure 3a). Fluorescence microscopy revealed that in inactivated endothelial cells without LPS stimulation, both Onex and V-Onex showed minimal DiI fluorescence, indicating a limited interaction under basal conditions. Similarly, Onex-treated cells under LPS stimulation exhibited weak fluorescence, suggesting low non-specific uptake under these conditions. In contrast, LPS-activated cells treated with V-Onex showed markedly increased DiI fluorescence localized along the cellular periphery and in the cytoplasm, indicating enhanced uptake under inflammatory conditions. The quantification of these fluorescence signals is presented in Supplementary Figure S2. These findings confirmed that V-Onex exhibits a preferential uptake profile toward inflamed endothelial cells. The selective fluorescence signal in V-Onex-treated inflamed endothelial cells, compared with the minimal uptake under other conditions, highlights the potential role of surface-displayed VHPK peptides in targeting. This targeted behavior is critical for improving therapeutic precision and reducing off-target distribution, supporting the application of V-Onex in inflammatory vascular disorders, such as atherosclerosis. To confirm that LPS stimulation effectively increased VCAM-1 expression, HUVECs were treated with 200 ng/mL of LPS for 24 h, followed by quantitative real-time PCR analysis (Figure 3b). The results demonstrated an approximately 150-fold increase in *VCAM-1* mRNA levels compared with those in untreated controls, indicating that inflamed HUVECs expressed sufficient receptors for V-Onex targeting. Although only mRNA levels were assessed in this study, previous reports have demonstrated a strong correlation between VCAM-1 gene expression and protein abundance under inflammatory conditions [45]. Future work will therefore include protein-level validation, such as immunofluorescence, to complement these findings.

### 2.5. Evaluation of the Anti-Inflammatory Gene Regulations of V-Onex in HUVECs and THP-1 Cells

To assess the anti-inflammatory potential of V-Onex, endothelial and immune cell models were evaluated under LPS-stimulated inflammatory conditions. HUVECs were cultured to confluence and stimulated with 200 ng/mL of LPS for 24 h to mimic the inflamed vascular endothelium. THP-1 monocytes were differentiated into M0 macrophages using PMA, followed by stimulation with 100 ng/mL of LPS for 24 h. After the induction of inflammation, both cell types were treated with Onex or V-Onex at a dose of 1 × 10^10^ particles/mL, and the expression levels of pro-inflammatory cytokines were assessed using qRT-PCR. In HUVECs, LPS stimulation markedly upregulated IL-1β and IL-6 expression, reflecting the activation of a pro-inflammatory endothelial phenotype. Onex treatment led to partial suppression of these markers, but V-Onex demonstrated a significantly greater inhibitory effect, reducing IL-1β and IL-6 expression levels by 32 and 44%, respectively, compared to those with LPS stimulation (Figure 3c). These results suggest that the targeted delivery capability of V-Onex facilitates enhanced vesicle internalization in inflamed endothelial cells, thereby increasing the local anti-inflammatory efficacy. VCAM-1 is strongly upregulated in endothelial cells in response to inflammatory signals, such as LPS; therefore, the VHPK peptide on the V-Onex surface likely facilitates receptor-mediated uptake, resulting in more effective suppression of pro-inflammatory cytokine expression. A similar inflammatory response was observed in THP-1 cells activated with 100 ng/mL of LPS (Figure 3d). The expression of IL-1β, TNF-α, and IL-6 was significantly elevated following LPS stimulation. Notably, both Onex and V-Onex effectively inhibited inflammatory gene expression in macrophages; however, unlike in endothelial cells, no statistically significant difference was observed between the two, whereas V-Onex retained the inhibitory effect observed with Onex. In particular, IL-6 expression was reduced by up to 45% compared to that with LPS stimulation, demonstrating substantial suppression of this pro-inflammatory cytokine. This suggests that V-Onex does not offer a targeting advantage in macrophages and instead exerts anti-inflammatory effects equivalent to those of unmodified Onex. This was likely due to the low VCAM-1 expression in THP-1 cells, resulting in non-specific vesicle uptake in both treatment groups. This result was fully expected. Considering in vivo application, V-Onex is intended to be taken up by macrophages to exert its anti-inflammatory effects. At the same time, its enhanced delivery to endothelial cells that overexpress VCAM-1 can lead to an increased therapeutic effect for atherosclerosis. Nevertheless, the preservation of anti-inflammatory activity following peptide modification indicates that surface engineering does not impair the intrinsic biological activity of vesicles. Collectively, these findings demonstrate that V-Onex maintains its anti-inflammatory activity in both endothelial and immune cells. Moreover, their ability to suppress cytokine expression more effectively in HUVECs reflects the benefits of receptor-targeted delivery. These features are particularly relevant in the context of atherosclerosis, in which inflamed endothelial cells at lesion sites actively regulate immune cell infiltration. V-Onex may serve as a site-specific anti-inflammatory nanoplatform that enhances therapeutic precision and reduces the off-target effects associated with systemic treatments.

### 2.6. Enhanced Suppression Activity of Endothelial Cell Migration by V-Onex

**Figure 4:**
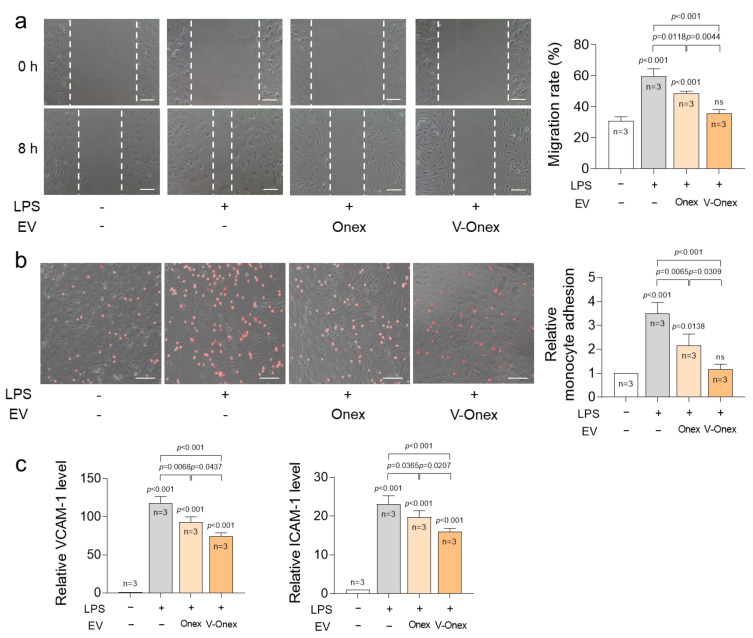
Inhibitory effects of V-Onex on endothelial cell migration, monocyte adhesion, and adhesion molecule expression. (**a**) Representative images and quantification of HUVEC migration assessed using a scratch assay with size bars indicating 100 µm. Cells were pretreated with LPS (200 ng/mL) for 24 h, followed by treatment with Onex or V-Onex (1 × 10^10^ particles/mL) for 8 h. (**b**) Representative images with size bars indicating 100 µm and quantification of monocyte adhesion to HUVECs. Cells were treated with LPS to induce VCAM-1 expression, followed by treatment with PBS, Onex, or V-Onex for 24 h. DiI-labeled THP-1 monocytes were co-incubated for 1 h, and adhesion was quantified. (**c**) Relative mRNA expression levels of VCAM-1 and ICAM-1 in LPS-stimulated HUVECs following treatment with Onex or V-Onex, analyzed using qRT-PCR. Data are presented as mean ± SEM. Statistical significance was determined using one-way ANOVA: ns: not significant. EV, extracellular vesicle; HUVEC, human umbilical vein endothelial cell; LPS, lipopolysaccharide.

In atherosclerosis, inflammation-driven endothelial migration contributes to the formation of intercellular gaps, thereby facilitating vascular permeability and immune cell infiltration of the intimal layer. This process accelerates lesion progression and destabilizes the vascular integrity, ultimately aggravating plaque development. Interfering with this process may attenuate the advancement of atherosclerotic plaques and maintain endothelial barrier function. To evaluate the effect of V-Onex on endothelial cell migration under inflammatory conditions, a wound-healing assay was performed using LPS-induced HUVECs. A linear scratch was generated to mimic a wound and the cells were treated with either Onex or V-Onex at 1 × 10^10^ particles/mL. Cell migration images were captured at 0 and 8 h, and gap closure was quantified using ImageJ software (ver.1.53t). As shown in Figure 4a, LPS stimulation led to rapid cell migration and wound closure, increasing the cell migration rate by approximately two-fold, from 30 to 60%. In contrast, the migration rate decreased to 18% upon Onex treatment, which was an 18% reduction compared with that in the untreated condition. The V-Onex treatment resulted in an even greater wound closure area, with a migration rate of 40% under the same conditions. This observation suggests that the targeting capability of V-Onex enhances its interaction with inflamed HUVECs, leading to a greater modulation of migration-associated signaling pathways. Several studies have linked VCAM-1 expression to leukocyte adhesion, cytoskeletal remodeling, and the migratory behavior of endothelial cells under inflammatory conditions [46,47]. The VHPK peptide on the surface of V-Onex is designed to bind VCAM-1, and this interaction may enhance vesicle internalization or membrane association, delivering anti-inflammatory cargo more efficiently, and thereby disrupting migratory signaling cascades. These findings suggest that V-Onex effectively limits the pathological endothelial dynamics under inflammatory conditions, potentially preventing the breakdown of barrier integrity and subsequent immune cell recruitment associated with atherosclerotic lesion formation.

### 2.7. Enhanced Suppression of Monocyte Adhesion to Endothelial Cells by VCAM-1-Targeting V-Onex

Monocyte adhesion to endothelial cells represents a critical early event in the pathogenesis of atherosclerosis, as it initiates vascular inflammation and facilitates the subsequent accumulation of immune cells within the intima. To assess the inhibitory effect of V-Onex on this process, a monocyte adhesion assay was performed using HUVECs stimulated with 200 ng/mL of LPS for 24 h to induce an inflamed endothelial phenotype. The cells were then treated with either Onex or V-Onex at a concentration of 1 × 10^10^ particles/mL. Adherent THP-1 monocytes were then quantified by fluorescence microscopy (Figure 4b). This model mimics the early inflammatory interaction between circulating monocytes and the vascular endothelium, enabling direct evaluation of the anti-adhesive capacity of EVs. LPS stimulation significantly increases monocyte adhesion to endothelial cells, reflecting an activated and adhesive endothelial phenotype. Treatment with Onex reduced monocyte adhesion by 38% compared with that in the LPS-only treated group, suggesting a partial anti-inflammatory effect. V-Onex further decreased the adhesion levels to 66% under the same conditions and achieved a statistically significant improvement over Onex. This effect indicates the enhanced suppression of inflammatory cell recruitment and highlights the superior anti-adhesive efficacy of V-Onex. This suggests that the targeting capacity of V-Onex facilitates more efficient modulation of adhesion-related signaling pathways in the inflamed endothelium. To elucidate the underlying mechanism, the mRNA expression levels of VCAM-1 and ICAM-1, two key adhesion molecules responsible for monocyte-endothelial cell interactions, were analyzed using qRT-PCR. These adhesion molecules play pivotal roles in leukocyte attachment and transendothelial migration, making them key therapeutic targets for modulating early inflammatory events. LPS treatment markedly increased the expression of both genes, with *VCAM-1* levels increasing 120-fold and ICAM-1 levels increasing 23-fold. Although Onex treatment reduced their expression to some extent, V-Onex treatment decreased VCAM-1 and ICAM-1 levels by 38 and 30%, respectively, representing a more pronounced reduction than the Onex treatment (Figure 4c). These findings indicated that V-Onex exerts a superior inhibitory effect on monocyte adhesion by downregulating the expression of endothelial adhesion molecules. Considering that monocyte recruitment is one of the earliest steps in atherosclerotic lesion formation, V-Onex may provide therapeutic benefits by intercepting this process at its origin. By targeting activated endothelial cells before immune cell infiltration, V-Onex can alter the plaque formation trajectory. Its ability to suppress endothelial activation and immune cell adhesion suggests that V-Onex may be an effective strategy for mitigating early vascular inflammation and preventing lesion progression during atherosclerosis.

### 2.8. Suppression of Foam Cell Formation by Onex and V-Onex Function

**Figure 5:**
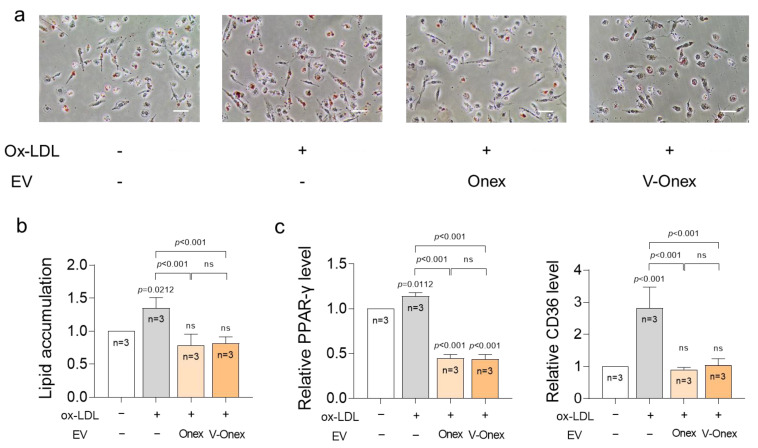
Inhibitory effects of V-Onex on foam cell formation in ox-LDL–stimulated macrophages. (**a**) Representative images with size bars indicating 100 µm and (**b**) quantification of intracellular lipid accumulation visualized by ORO staining in macrophages. (**c**) Relative mRNA expression levels of PPAR-γ and CD36 in THP-1 cells stimulated with ox-LDL (50 μg/mL) and treated with and without EVs, Onex, or V-Onex (1 × 10^10^ particles/mL) for 24 h, analyzed using qRT-PCR. Data are presented as mean ± SEM. Statistical significance was determined using one-way ANOVA: ns: not significant. EV, extracellular vesicle; ORO, Oil Red O, ox-LDL, oxidized low-density lipoprotein.

To evaluate whether Onex and V-Onex suppress foam cell formation, Oil Red O (ORO) staining was performed to visualize and quantify intracellular lipid accumulation. Ox-LDL stimulation of THP-1 macrophages resulted in a notable increase in lipid accumulation, as evidenced by the presence of numerous lipid-laden macrophages in the microscopic images. Treatment with either Onex or V-Onex visibly reduced the lipid staining intensity in the images, indicating the attenuation of foam cell formation (Figure 5a). First, ox-LDL treatment increased lipid accumulation in macrophages by 1.4-fold; however, both Onex and V-Onex significantly reduced ORO signal intensity compared to the ox-LDL-only treated group by 45% and 46%, respectively. No statistical difference was observed between the two EV-treated groups because of the low level of VCAM-1 in the macrophages, as mentioned above (Figure 5b). This result confirmed the ability of both vesicles to suppress lipid accumulation. Since foam cells also have very low expression of VCAM-1, a targeting effect by V-Onex cannot be achieved. Thus, this result also confirms that V-Onex, like Onex, maintains the inhibitory effect on foam cell formation, and therefore, it can inhibit one of the various phenomena that occur in an atherosclerotic environment. To further validate these results at the transcriptional level, qRT-PCR was performed to measure the expression of foam cell-associated markers (PPARγ and CD36). PPARγ and CD36 are well-established markers of foam cell formation. Macrophages were stimulated with ox-LDL, and the expression of foam cell-associated markers was analyzed. Cells were treated with ox-LDL in the presence or absence of EVs (1 × 10^10^ particles/mL), and mRNA levels of PPARγ and CD36 were measured. Ox-LDL stimulation significantly increased the expression of both PPARγ and CD36. Treatment with either Onex or V-Onex showed no notable difference in their effects, with both reducing PPARγ by 61% and CD36 by 65% on average compared with ox-LDL stimulation (Figure 5c). These results confirmed that both Onex and V-Onex effectively suppressed foam cell formation in macrophages by inhibiting lipid uptake and intracellular accumulation, despite the absence of targeted delivery in this cell type. Although V-Onex has demonstrated superior performance in VCAM-1-expressing endothelial models, its behavior in macrophages mirrors that of native Onex, reinforcing the idea that surface engineering does not compromise intrinsic vesicle function. The shared efficacy observed in macrophages highlights the potential of both vesicles to modulate atherogenic processes independent of their targeting capability.

In this study, developed a candidate therapeutic drug for atherosclerosis using Onex, a plant-derived EVs with potent anti-inflammatory properties. Specifically, we constructed V-Onex, in which Onex is conjugated with the VHPK peptide, which targets VCAM-1 and is overexpressed in endothelial cells in inflammatory environments. V-Onex exhibits multifunctional therapeutic effects in multiple in vitro models by significantly reducing the expression of pro-inflammatory cytokines, suppressing endothelial cell migration, decreasing monocyte adhesion, and attenuating foam cell formation. These effects collectively demonstrate the capacity of V-Onex to regulate key pathological processes involved in atherosclerosis. Although the reductions in cytokine expression may appear modest in in vitro experiments, these changes were consistently accompanied by functional outcomes such as suppression of endothelial cell migration, inhibition of monocyte adhesion, and reduction in foam cell formation. These processes are expected to coexist and act synergistically in in vivo conditions, thereby amplifying the therapeutic efficacy of V-Onex. Compared with conventional treatments, V-Onex offers several distinct advantages. Its plant origin ensures high biocompatibility and scalability, whereas surface engineering confers targeting specificity, which enhances its efficacy at lower dosages. The simplicity of this engineering strategy, involving membrane insertion facilitated by DSPE conjugation, supports the feasibility of translational development. In contrast to approaches that address single disease pathways, V-Onex has a multifaceted therapeutic effect by modulating vascular inflammation, immune cell recruitment, and lipid handling in macrophages. Together, these findings suggest that V-Onex represents not only a potential therapeutic agent for atherosclerosis but also a modular nanoplatform that can be adapted for other inflammatory vascular disorders.

## 3. Materials and Methods

### 3.1. Cell Cultures

HUVECs were maintained in Medium 200 (Cat. No. M200500, Thermo Fisher Scientific, Waltham, MA, USA), supplemented with Low Serum Growth Supplement (Cat. No. S00310, Thermo Fisher Scientific, Waltham, MA, USA) and 1% (*v*/*v*) penicillin-streptomycin (Cat. No. 15140122, Gibco, Waltham, MA, USA). Human monocytes (THP-1 cells) were cultured in RPMI-1640 medium containing 10% (*v*/*v*) FBS (Gibco) and 1% (*v*/*v*) penicillin-streptomycin (Gibco). All cells were incubated at 37 °C in a humidified incubator with 5% CO_2_. LPS (Cat. No. 93572-42-0, Sigma-Aldrich, St. Louis, MO, USA) was used for inflammatory stimulation, whereas foam cell formation was induced by treatment with ox-LDL (Cat. No. 360-31, Lee Biosolutions, Maryland Heights, MO, USA).

### 3.2. Isolation of Extracellular Vesicles from Onion

Onex were isolated from *Allium cepa* purchased from a local market in Korea using a sequential procedure involving blending, centrifugation, ultrafiltration, and size-exclusion chromatography (Cat. No. ICO-70, Izon Science, Christchurch, New Zealand).

### 3.3. NTA and Zeta Potential Analysis

Particle size distribution and Onex concentration were measured using a NanoSight NS300 instrument (Malvern Panalytical Ltd., Malvern, UK). Zeta potential and PDI were determined using a Zetasizer Nano ZS (Malvern Panalytical Ltd., Malvern, UK).

### 3.4. Effects of Onex on Cell Viability

To assess cell viability, HUVECs and THP-1 cells were seeded into 96-well plates and incubated for 24 h. Cells were then treated with Onex or V-Onex at concentrations ranging from 1.0 × 10^8^ to 1.0 × 10^10^ particles/mL for 72 h. Cell viability was measured using trypan blue exclusion assay.

### 3.5. qRT-PCR Analysis

qRT-PCR was performed using the StepOnePlus Real-Time PCR System (Applied Biosystems, Foster City, CA, USA) to evaluate the effect of Onex on inflammation-related gene expression. Total RNA was extracted with FavorPrep Tri-RNA Reagent (FAVORGEN, Ping-Tung, Taiwan) following the manufacturer’s instructions. Complementary DNA was synthesized using ReverTra Ace qPCR RT Master Mix (Toyobo, Osaka, Japan) and amplification was performed using THUNDERBIRD SYBR qPCR mix (Toyobo, Japan). Primer sequences used in this study are listed in Table S1.

### 3.6. Construction of V-Onex

To modify the surface of Onex with the VHPK peptide, DSPE-PEG-VHPK conjugates were synthesized. FITC-labeled VHPK peptide (VHPKQHRGGSKGC) was purchased from Peptron (Daejeon, Republic of Korea). Conjugation was performed using EDC/NHS chemistry, in which the carboxyl group of DSPE-PEG-COOH (1,2-distearoyl-sn-glycero-3-phosphoethanolamine-polyethylene glycol) was coupled to the amine group of the peptide. DSPE-PEG2000-COOH was dissolved at 5 mg/mL in MES buffer, followed by the addition of EDC and sulfo-NHS in a 1:1 molar ratio in MES buffer for 15 min. The peptide (10 mg/mL) was then added and incubated for 2 h. The resulting solution was dialyzed against ultrapure water for 3 days using a 2 kDa MWCO dialysis membrane (Spectra/Por, Thermo Fisher Scientific), and the final product was lyophilized and stored at −20 °C. For surface functionalization, the DSPE-PEG–VHPK conjugate was incubated with Onex at 37 °C for 4 h under gentle stirring, and unbound conjugates were removed by ultrafiltration. The resulting V-Onex was subsequently used for characterization and further experiments.

### 3.7. Targeting Effect Assessment of V-Onex

To evaluate the targeting efficiency of V-Onex, both Onex and V-Onex were labeled with fluorescent dye DiI and incubated with HUVECs. Following treatment, the cells were cultured in the presence or absence of the labeled vesicles. Cellular uptake was qualitatively assessed using fluorescence microscopy (Eclipse Ti2, Nikon, Tokyo, Japan).

### 3.8. Monocyte Adhesion Assay

HUVECs were seeded at a density of 2 × 10^4^ cells/cm^2^ in 24-well plates for microscopy-based quantification. All experiments were conducted using confluent HUVECs. Inflammation was induced by treatment with LPS for 24 h, followed by treatment with Onex or V-Onex. THP-1 cells were fluorescently labeled with DiI (1,1′-dioctadecyl-3,3,3′,3′-tetramethylindocarbocyanine perchlorate; cat# D282, Thermo Fisher Scientific) by incubation at 37 °C for 20 min. The labeled THP-1 cells were then added to the endothelial monolayers at a density of 5 × 10^4^ cells/well and incubated for 1 h at 37 °C in a 5% CO_2_ atmosphere. After incubation, unbound monocytes were removed by washing twice with PBS. Adherent THP-1 cells were observed under a fluorescence microscope.

### 3.9. Migration Assay

HUVECs were seeded in 24-well plates at a density of 2 × 10^4^ cells/cm^2^ and cultured for 24 h until they reached near-confluence. Inflammation was induced by treating the cells with LPS (Sigma-Aldrich), followed by incubation with either Onex or V-Onex. A mechanical wound was created by scratching the monolayer with a 200-μL pipette tip and detached cells were removed by washing twice with PBS. After 8 h of incubation, images of cell migration into the scratch area were captured using an Eclipse TS2 inverted microscope (Nikon) and analyzed using the ImageJ software (National Institutes of Health, Bethesda, MD, USA, ver.1.53t). Migration rate was calculated using the following equation:

Migration rate (%) = [(A_0_ − A_t_)/A_0_] × 100,

where A_0_ represents the area of the scratch at time 0 and A_t_ is the area at each subsequent time point.

### 3.10. ORO Staining

ORO staining was used to assess lipid accumulation in the macrophages. ORO powder (Sigma-Aldrich) was dissolved in isopropyl alcohol and filtered through a 0.2-μm syringe filter (GVS, Zola Predosa, Italy) before use. Cells were fixed with formalin and stained with ORO solution to visualize intracellular lipids. After microscopic observation, the stained lipids were eluted with isopropanol, and absorbance was measured at 450 nm using a Varioskan Flash Multimode Reader (Thermo Fisher Scientific) to quantify lipid content.

### 3.11. Statistical Analysis

The results are expressed as mean ± standard deviation, *n* ≥ 3. Data analysis was performed using one-way ANOVA, with a *p*-value of less than 0.05 denoting statistical significance. All statistical evaluations were derived from independent experiments and performed using GraphPad Prism 7.0 software (GraphPad, La Jolla, CA, USA).

## 4. Conclusions

In this study, we demonstrated that engineered V-Onex, which can target VCAM-1-expressing endothelial cells, possesses excellent biocompatibility and stability, and exhibits no cytotoxicity in endothelial cells and macrophages. V-Onex effectively and selectively accumulated in the inflamed endothelial cells, leading to a significant reduction in the expression of inflammatory markers in both cell types. Furthermore, V-Onex suppressed the migration of endothelial cells and reduced their interaction with monocytes, both of which are critical processes that contribute to atherosclerotic plaque formation. Importantly, in THP-1 cells, V-Onex inhibited the uptake of ox-LDL and consequently reduced foam cell formation. Collectively, these compelling results highlight V-Onex as a highly promising and modular targeted nanovesicle platform capable of modulating multiple pathological processes associated with atherosclerosis. However, the present study is limited to in vitro validation and did not include pharmacological controls such as NSAIDs. Plant-derived EVs are inherently heterogeneous, and large-scale production and standardization remain challenges for clinical translation. Future research should incorporate in vivo biodistribution and efficacy studies to confirm these therapeutic effects. Also, unlike cell-derived EVs, information about the components of plant EVs is quite limited. To develop a therapeutic agent using plant EVs, it is necessary to identify the components encapsulated within them. Additional research to identify the components loaded onto Onex, such as miRNA, lipids, metabolites, and proteins, is expected to significantly contribute to the development of V-Onex as a new targeted therapeutic agent for atherosclerosis.

## Data Availability

The original data supporting the conclusions of this study are included in the article and its Supplementary Materials. Further inquiries can be directed to the corresponding author.

## Supplementary Materials

The supporting information can be downloaded at: https://www.mdpi.com/article/10.3390/ijms26188884/s1.

## Abbreviations

The following abbreviations are used in this manuscript:

CVD	Cardiovascular disease
EV	Extracellular vesicle
LPS	Lipopolysaccharide
NTA	Nanoparticle tracking analysis
ORO	Oil Red O
Ox-LDL	Oxidized low-density lipoprotein
PDI	Polydispersity index
