# Supplementary material for: Targeted Atherosclerosis Treatment Using Vascular Cell Adhesion Molecule-1 Targeting Peptide-Engineered Plant-Derived Extracellular Vesicles
Source: Int J Mol Sci. 2025 Sep 12;26(18):8884. doi: 10.3390/ijms26188884 (PMC12470028; doi:10.3390/ijms26188884)
Supplement: Supplementary file 1 [file ijms-26-08884-s001.zip › ijms-3858399-supplementary.pdf]

Supplementary information

Targeted atherosclerosis treatment using vascular cell adhesion molecule-1 targeting peptide-engineered plant-derived extracellular vesicles

Chanwoo Choi[1], Won Jong Rhee[1,2,3] *

AUTHOR ADDRESS

[1] Department of Bioengineering and Nano-Bioengineering, Incheon National University, Incheon 22012, Republic of Korea

[2] Division of Bioengineering, Incheon National University, Incheon 22012, Republic of Korea

[3] Research Center for Bio Materials & Process Development, Incheon National University, Incheon 22012, Republic of Korea

[*] To whom correspondence should be addressed. Tel.: +82 32 835 8299; Fax: +82 32 835 0763; Email: wjrhee@inu.ac.kr

Supplementary Table and Figure

Table S1. Primer sequence for qRT-PCR analysis

Primer	Sequences (5' - 3’)
Human β-actin F	ATGAAGTGTGACGTTGACATCCG
Human β-actin R	GCTTGCTGATCCACATCTGCTG
Human IL-1β F	AGCTGATGGCCCTAAACAGA
Human IL-1β R	TCGGAGATTCGTAGCTGGAT
Human IL-6 F	GTGTGAAAGCAGCAAAGAG
Human IL-6 R	GGGCAAGTCTCCTCATTGAATCC
Human TNF-α F	CCCCAGGCAGTCAGATCATCTTCT
Human TNF-α R	ATGAGGTACAGGCCCTCTGAT
Human VCAM-1 F	CCCTTGACCGGCTGGAGATT
Human VCAM-1 R	CTGGGGGCAACATTGACATTAAGTG
Human ICAM-1 F	GGCCACGCATCTGATCTGTA
Human ICAM-1 R	ACTTCCCCTCTCATCAGGCT

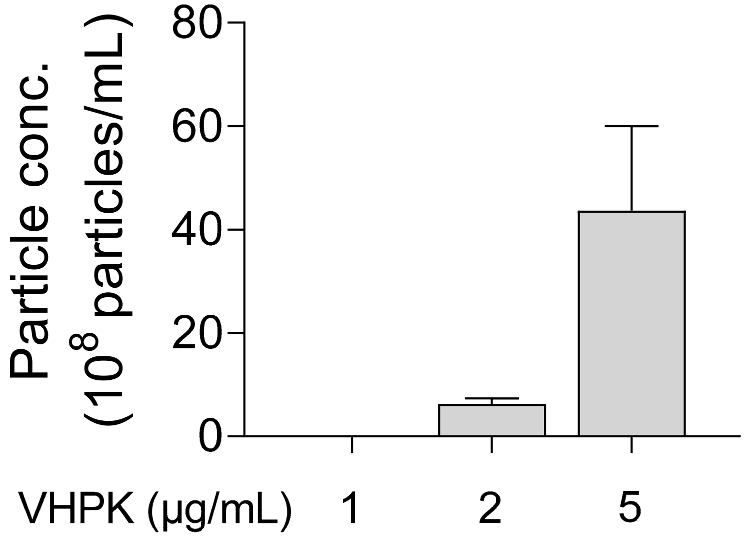

Figure S1. Particle concentration measured after incubating DSPE-PEG-VHPK at different concentrations (1, 2, and 5 μg/mL) in PBS, followed by ultrafiltration. Micelle formation was evident at concentrations above 2 μg/mL, whereas no micelle formation was detected at 1 μg/mL. Based on these findings, 1 μg/mL was selected as the optimal peptide concentration for subsequent V-Onex synthesis to prevent micelle formation.

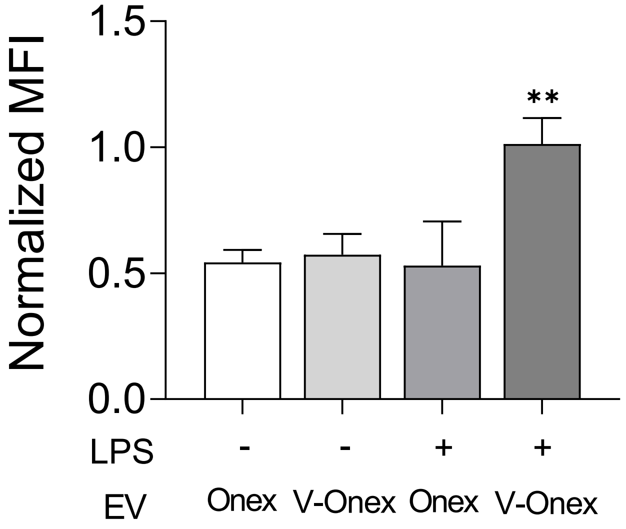

Figure S2. Quantification of V-Onex uptake in HUVECs. HUVECs were incubated with DiI-labeled Onex or V-Onex (1 × 10^{10} particles/mL) in the presence or absence of LPS stimulation. Cellular uptake of EVs was visualized by fluorescence microscopy, and normalized mean fluorescence intensity (MFI) was calculated as the ratio of red fluorescence (EVs) to blue fluorescence (nuclei) using ImageJ. Data are presented as mean ± SEM (n = 3).
